# MECHANISMS OF COGNITIVE AND NEUROLOGICAL AGING

**DOI:** 10.1093/geroni/igz038.2176

**Published:** 2019-11-08

**Authors:** Catherine Kaczorowski

**Affiliations:** The Jackson Laboratory, Bar Harbor, Maine, United States

## Abstract

This session will focus on recent advances in understanding how biological aging impacts neurological function and risk of developing age-related neurodegenerative diseases.

